# Pulmonary Hypertension in Association with Lung Disease: Quantitative CT and Artificial Intelligence to the Rescue? State-of-the-Art Review

**DOI:** 10.3390/diagnostics11040679

**Published:** 2021-04-09

**Authors:** Krit Dwivedi, Michael Sharkey, Robin Condliffe, Johanna M. Uthoff, Samer Alabed, Peter Metherall, Haiping Lu, Jim M. Wild, Eric A. Hoffman, Andrew J. Swift, David G. Kiely

**Affiliations:** 1 Department of Infection, Immunity and Cardiovascular Disease, University of Sheffield, Sheffield S10 2RX, UK; michael.sharkey3@nhs.net (M.S.); robin.condliffe@nhs.net (R.C.); s.alabed@sheffield.ac.uk (S.A.); peter.metherall@nhs.net (P.M.); J.M.Wild@sheffield.ac.uk (J.M.W.); A.J.Swift@sheffield.ac.uk (A.J.S.); david.kiely1@nhs.net (D.G.K.); 2 Radiology Department, Sheffield Teaching Hospitals NHS Foundation Trust, Sheffield S10 2JF, UK; 3 Sheffield Pulmonary Vascular Disease Unit, Royal Hallamshire Hospital, Sheffield Teaching Hospitals NHS Foundation Trust, Sheffield S10 2JF, UK; 4 Department of Computer Science, University of Sheffield, Sheffield S1 4DP, UK; j.uthoff@sheffield.ac.uk (J.M.U.); h.lu@sheffield.ac.uk (H.L.); 5 INSIGNEO, Institute for In Silico Medicine, University of Sheffield, Sheffield S1 3JD, UK; 6 Advanced Pulmonary Physiomic Imaging Laboratory, University of Iowa, C748 GH, Iowa City, IA 52242, USA; eric-hoffman@uiowa.edu

**Keywords:** pulmonary hypertension, PH-Lung disease, quantitative CT, hypoxia, artificial intelligence, machine learning, pulmonary arterial hypertension

## Abstract

Accurate phenotyping of patients with pulmonary hypertension (PH) is an integral part of informing disease classification, treatment, and prognosis. The impact of lung disease on PH outcomes and response to treatment remains a challenging area with limited progress. Imaging with computed tomography (CT) plays an important role in patients with suspected PH when assessing for parenchymal lung disease, however, current assessments are limited by their semi-qualitative nature. Quantitative chest-CT (QCT) allows numerical quantification of lung parenchymal disease beyond subjective visual assessment. This has facilitated advances in radiological assessment and clinical correlation of a range of lung diseases including emphysema, interstitial lung disease, and coronavirus disease 2019 (COVID-19). Artificial Intelligence approaches have the potential to facilitate rapid quantitative assessments. Benefits of cross-sectional imaging include ease and speed of scan acquisition, repeatability and the potential for novel insights beyond visual assessment alone. Potential clinical benefits include improved phenotyping and prediction of treatment response and survival. Artificial intelligence approaches also have the potential to aid more focused study of pulmonary arterial hypertension (PAH) therapies by identifying more homogeneous subgroups of patients with lung disease. This state-of-the-art review summarizes recent QCT developments and potential applications in patients with PH with a focus on lung disease.

## 1. Introduction

**Figure 1:**
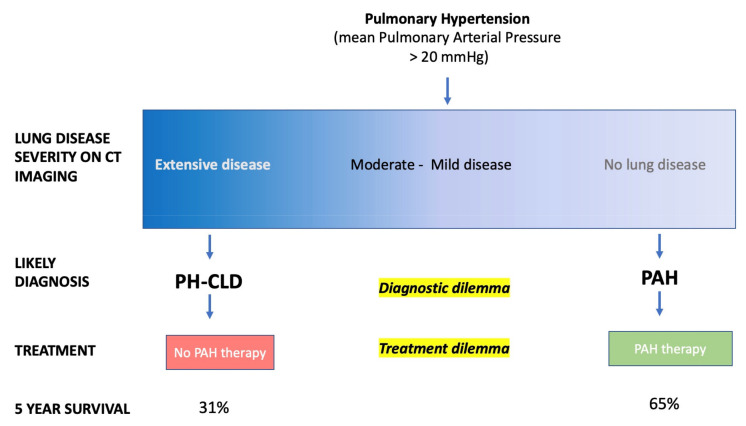
Spectrum of lung disease severity within pulmonary hypertension and the diagnostic and treatment dilemma. Five year survival figures quoted from REVEAL (Registry to Evaluate Early and Long-Term PAH Disease Management) registry five year outcomes and recent studies [3,18,19].

Pulmonary hypertension (PH) is a heterogeneous, life-limiting condition defined by an elevated pulmonary artery pressure and, if untreated, results in right ventricular failure and death. The current classification of PH identifies five groups, each with shared pathophysiological characteristics [1]. However, increasingly, patients are identified with overlapping features. Group 3 PH—pulmonary hypertension in association with lung disease and/or hypoxia (PH-Lung)—is a complex, highly heterogeneous group where pre-capillary PH most commonly complicates chronic obstructive pulmonary disease (COPD) and/or emphysema, interstitial lung disease (ILD), and alveolar hypoventilation [2,3,4,5]. These conditions may also co-exist to varying degrees, and patients with a combination of pulmonary fibrosis and emphysema (CPFE) with severe PH have a particularly poor prognosis [3]. Usually, patients with PH in association with lung disease have a mild elevation of pulmonary artery pressure in keeping with the severity of the underlying lung disease, however, some patients present with severe PH with variable degrees of parenchymal involvement [5]. A review by Kovacs et al. highlighted the spectrum of pulmonary vascular involvement in patients with COPD [6]. In these patients, it is important to exclude other forms of PH, such as Group 1 PH, pulmonary arterial hypertension (PAH), and group 4 PH (chronic thromboembolic pulmonary hypertension (CTEPH)) [7]. Idiopathic pulmonary arterial hypertension (IPAH) is a form of PAH where no other cause of PH is identified, although, in some cases, it has a heritable component [8,9,10]. Current guidelines recommend that patients with severe pre-capillary PH with no other cause identified be classified as Group 1 IPAH [5,11], whereas patients with severe lung disease and mild PH are classified as having Group 3 PH-Lung. However, in clinical practice, some patients do not neatly fit into either category, and this creates a clinical dilemma (Figure 1).

The recommended managements of IPAH and PH-Lung are divergent. The last decade has seen the introduction of multiple novel PAH specific drug therapies targeting endothelin, nitric oxide, and prostanoid pathways [12]. These have improved the outlook for PAH, particularly in younger patients with IPAH where the UK National Audit reports a 5 year survival in excess of 80% [13]. Previous data from the ASPIRE ) Assessing the Spectrum of Pulmonary Hypertension Identified at a Referral Centre) Registry highlighted the impact of lung disease on survival compared with other forms of PH. Patients with PH-Lung assessed at a PH referral center had a 5 year survival of 31%, worse than all other forms of PH [14]. For patients with severe PH-Lung, the survival is particularly poor, worse in ILD compared to COPD [14]. In contrast to Group 1 PAH, for patients with Group 3 PH-Lung, guidelines recommend treatment of the underlying lung disease or, in highly selected cases, lung transplantation. Interestingly, the sixth World Symposium on PH (WSPH) recommended that patients with minor lung disease, who otherwise meet the criteria for PAH with no other causes identified, should be considered to have IPAH [5]. This recommendation was based on unpublished subgroup analysis in patients with mild to moderate lung disease from randomized controlled studies [15,16,17]. However, a recent publication demonstrated that patients diagnosed as IPAH using criteria from the sixth WSPH with mild lung disease had significantly worse survival with no improvement in exercise capacity or quality of life [11]. This study highlights the challenge of classifying patients with PH and a need for improved disease phenotyping to ensure that the most appropriate patients receive treatment and, where uncertainty exists, that patients are entered into appropriate clinical trials.

**Figure 2:**
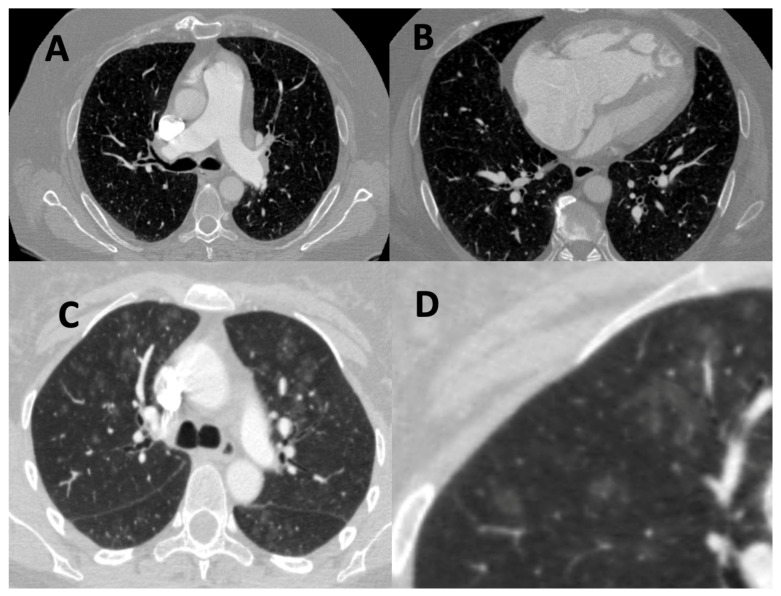
Computed tomography (CT) features of pulmonary arterial hypertension (PAH) on CT. (**A**) Dilated main pulmonary artery. (**B**) Right atrial and ventricular dilation with moderate right ventricular hypertrophy and flattening of the interventricular septum. (**C**) Centrilobular ground glass nodularity. These are a feature of PAH but are also more commonly seen in another sub-phenotypes of pulmonary hypertension, such as pulmonary vascular obstructive disease (PVOD). In PVOD, they are often accompanied by interlobular septal thickening and mediastinal lymphadenopathy. (**D**) Zoomed in view of regions of centrilobular ground glass nodularity.

**Figure 3:**
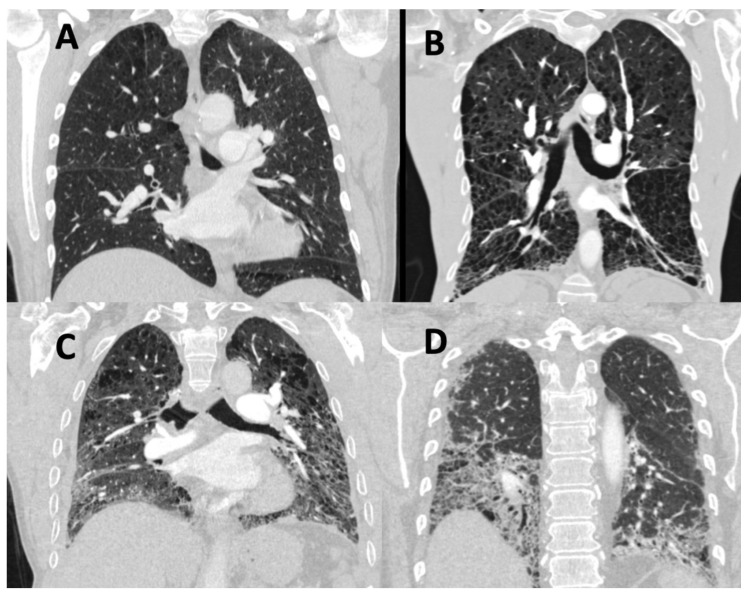
Patterns of lung disease in pulmonary hypertension in association with lung disease and/or hypoxia (PH-Lung) as visualized on CT. (**A**) Mild emphysema localized predominantly to the upper lobe. (**B**) Widespread severe emphysema. (**C**) Combined emphysema and fibrosis. (**D**) Interstitial lung disease.

Traditionally, the severity of lung disease is quantified using lung function tests using spirometric values, lung volumes, and measurements of gas diffusion (DLco and Kco), with increasing evidence showing that DLco is strongly prognostic in a number of forms of PH [20,21,22]. Indeed, DLco is part of a widely used risk score, REVEAL (Registry to Evaluate Early and Long-Term PAH Disease Management) 2.0 [23]. Lung function tests, however, show significant variability, low reproducibility, and can be insensitive to change to disease progression [24,25,26]. Computed tomography (CT) is the gold standard for evaluating the extent and the distribution of lung parenchymal disease and provides an additional non-invasive assessment of vascular, cardiac, and mediastinal structures [27,28]. CT is, therefore, recommended by both the latest joint European Respiratory Society/European Society of Cardiology PH guidelines and the Pulmonary Vascular Research Institute (PVRI) statement on imaging in pulmonary hypertension as an important part of the diagnostic strategy in suspected PH [1,29]. The features and the patterns of disease in PAH and PH-Lung on CT are visualized in Figure 2 and Figure 3, respectively.

Artificial intelligence (AI) has seen a rise in prominence and performance, especially with recent machine learning (ML) approaches. It has been labeled as “a major paradigm shift” and “one of the most fundamental changes in medical care” [30,31]. Within chest CT and respiratory medicine, there has been an explosion in AI research, with a 5× increase in studies between 2014–2019 compared to 2010–2014. Correspondingly, there are now >75 Food and Drug Administration (FDA) and several CE approved AI software packages in chest CT alone compared to just one in 2014 [32]. We reviewed the literature and found no studies directly involving AI, chest CT, and PH. There are, however, many studies involving AI, ML, and chest CT in other diffuse lung diseases such as emphysema, COPD, ILD, and, recently, COVID-19. Discoveries, solutions, and findings of these studies are directly applicable to development of AI models in PH.

Despite a number of studies highlighting the negative impact of PH on survival in PH-Lung [2,7,33], data are very limited for the use of PAH therapies. Studies have been performed primarily in COPD and ILD and yielded conflicting results [5]. This reflects, in part, the small numbers, the study designs, and the heterogeneous nature of patients enrolled. Despite studies with ambrisentan [34] and riociguat [35] identifying safety concerns in patients with ILD a recent randomized controlled study, INCREASE demonstrated an improvement in the 6 minute walk test in patients with ILD [36]. This has re-invigorated the PH community to explore PAH therapies in PH-Lung. However, given the heterogenous nature of PH in lung disease, a technique to aid the identification of more homogeneous subgroups of PH-Lung would be very welcome.

Given the potential of AI approaches to assess lung parenchyma, vessels, and cardiac chambers, the authors postulate that quantitative CT (QCT) could “come to the rescue” of investigators wishing to improve the outlook for patients with PH-Lung. By improving phenotyping, AI approaches could aid the characterization of disease and enrich populations most likely to benefit from PAH therapies. This state-of-the-art review critically appraises the recent developments in the adjacent fields of ML in CT imaging and contextualizes their potential impact on imaging, diagnosis, classification, and assessment of prognosis in patients with PH with a focus on lung disease.

## 2. AI, Machine Learning and Deep Learning

### 2.1. Definitions

**Figure 4:**
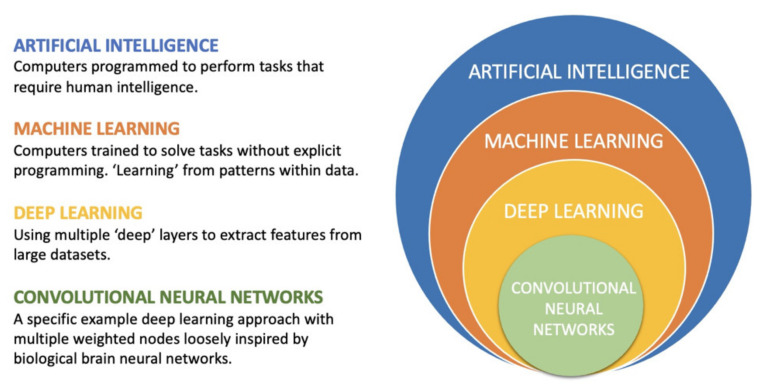
Layers of artificial intelligence approaches applied to medical imaging.

Artificial intelligence (AI) is a general term encompassing computer algorithms capable of performing tasks requiring human intelligence. Figure 4 summarizes the relationship between different AI approaches. Historically, AI has been trained with a rules-based approach, where a programmer explicitly creates a set of conditions upon which the machine executes actions. 

Machine learning (ML) is a subset of AI in which algorithms are trained to solve tasks through feature learning instead of an explicit rules-based approach. When presented with a “training” cohort, the algorithm identifies salient features, which are subsequently used to make predictions. Hence, the “machine” “learns” from the data itself.

Deep learning (DL) is a subset of ML which uses multiple layers to extract features. Each “layer” provides information to the algorithm. “Convolutional neural networks” (CNNs) are a subset and one approach to “deep learning”, which is loosely inspired by neurons in the human brain. Several “nodes” exist within multiple layers, and each “node” as a data point has a weighting which affects the whole “network”. This approach in particular is most prevalent in the domain of image recognition and analysis and has demonstrated superior problem-solving capabilities. While most earlier AI methods have led to applications with subhuman performance, recent deep learning algorithms are able to match and even surpass humans in task-specific applications [30,37,38,39,40,41]. Two recent examples of AI superiority in medical imaging include measuring wall thickness in hypertrophic cardiomyopathy on cardiac magnetic resonance imaging (MRI) and diagnosing breast cancer in mammography [37,42].

### 2.2. Supervised vs Unsupervised Learning

Two broad categories of ML methods exist—supervised and unsupervised learning. Supervised learning requires explicit labeling of the training data with a ground truth, upon which the machine develops its model and makes predictions. Labeling lung parenchymal disease patterns as “ground glass change” or “emphysema” is an example of supervised learning. Performance is tested by how well it predicts these labels in a validation cohort. Unsupervised learning requires no explicit labeling of the training data; rather, it learns to cluster/group together, thereby requiring very large datasets [43]. The algorithm makes its own inferences within the data space to learn its internal structure and uses that make predictions. Semi-supervised learning is a combination of two methods where a smaller labeled dataset is combined with a larger unlabeled dataset. The labeled dataset is used to guide the algorithm before it is used in the unlabeled data. The vast majority of all medical imaging AI studies use supervised learning and are therefore reliant on accurate labeling.

### 2.3. General vs. Narrow Intelligence

General intelligence is the ability to apply knowledge across a range of domains. A radiologist is trained to have “general” intelligence across all modalities and age groups, from detecting congenital anomalies in fetal ultrasounds to complex neurodegenerative disease in brain MRIs. Methods and skills learned in one domain are often transferable to other domains. In contrast, almost all state-of-the-art AI advances are currently limited to narrow intelligence in a defined domain [30]. The focus of such studies has been to match and occasionally surpass human radiologist performance in that narrow specific instance. How “narrow” an algorithm is depends on how it was developed and what datasets were used; an algorithm trained entirely on ILD could not evaluate emphysema.

**Figure 5:**
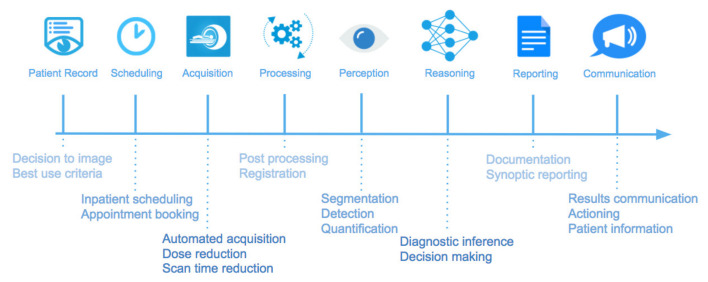
Stages within a radiology diagnostic workflow with potential artificial intelligence (AI) applications at each stage. This review focuses on the image analysis stage, incorporating image perception and reasoning. Image reproduced with permission from original author Dr. Hugh Harvey [44].

Figure 5 outlines the multiple stages within a diagnostic radiology workflow, highlighting the potential avenues for AI solutions. This review focuses on the image perception and reasoning stages, however, each stage is a current active research topic of interest.

## 3. Machine Learning in Chest CT

Within image analysis, machine learning in chest CT broadly has two domains: nodule detection and radiomics. Multiple AI solutions exist for lung cancer nodule detection and have shown to have a high level of accuracy, sensitivity, and specificity [45,46,47]. Labeled public datasets for model development exist, the largest of which is the Lung Image Database Consortium Image Collection (LIDC-IDRI) [48]. Radiomics is the broad field of study which aims to extract information from imaging using computer-aided mathematical analysis that is not accessible through traditional visual inspection [49]. Initial use was predominantly in oncology settings to make predictions on disease course, survival, and treatment response from tumor features [49,50]. Recently, the field has expanded to non-oncological settings. In diffuse lung diseases, QCT has been the main application of ML algorithms.

**Figure 6:**
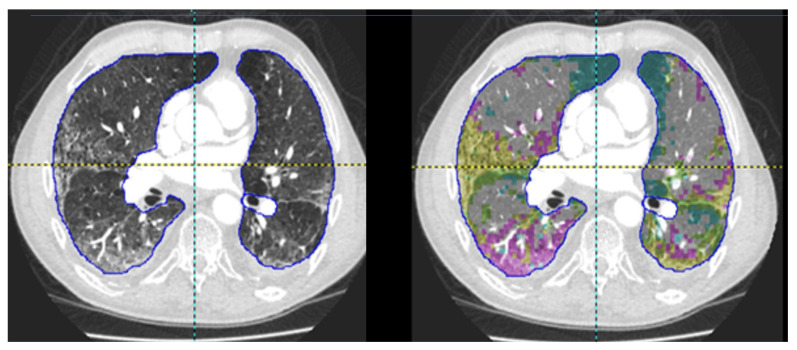
Demonstration of a quantitative CT (QCT) approach (adaptive multiple features method), acquired using PASS software. Different lung parenchymal disease patterns are identified and highlighted. Blue, emphysema/low attenuation pattern. Yellow, fibrotic changes. Pink, ground glass change.

Quantitative CT (QCT) is the principle of extracting quantitative information from standardized imaging data. This ranges from simple human hand-drawn manual measurements of anatomical structures such as the trachea or the main pulmonary artery (mPA) to complex AI driven texture analysis of lung parenchymal disease patterns. It has been used in a range of diffuse lung diseases [51,52]. State-of-the-art applications of QCT use ML and DL to provide end-to-end solutions, where the entire CT scan is automatically analyzed with an output provided. Such approaches require a suite of algorithms, from segmentation where the lung parenchyma is accurately identified and extracted to quantification and incorporation into clinical models. A recent example is CORADS-AI, which automatically quantifies and assigns the CORADS (COVID-19 Reporting and Data System) score for the extent of lung parenchymal in COVID-19 [53]. Figure 6 demonstrates such a QCT approach using an adaptive multiple features method with different parenchymal patterns highlighted [54].

## 4. Promise of Quantitative Chest CT in PH

The current standard for assessment of chest CT is by an expert radiologist. This approach fundamentally treats scans as pictures for subjective visual assessment. QCT and AI approaches, in contrast, treat scans as imaging data, which can be processed and analyzed.

**Figure 7:**
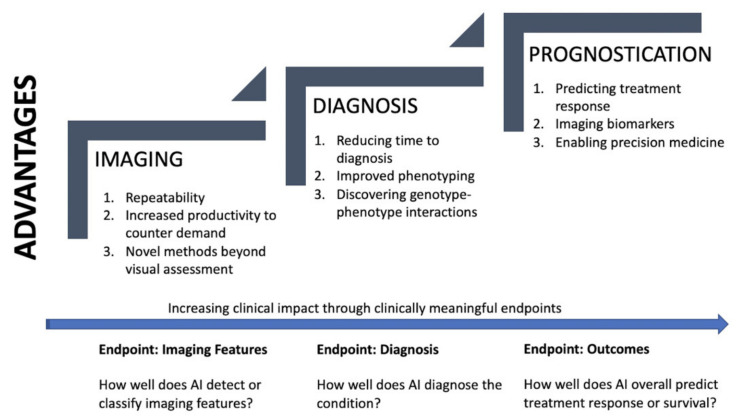
Summary figure. Domains of AI application with corresponding advantages. Increasing clinical impact through clinically meaningful endpoints.

AI models can be categorized by their domain of application and corresponding endpoints. Figure 7 is a summary diagram which visualizes the promise and the advantages of QCT in each domain—imaging, diagnosis, and prognostication.

### 4.1. Imaging

#### 4.1.1. Repeatability

Radiological assessment is a subjective process. Reports between radiologists differ significantly in their style and content, and there exists significant inter-observer variability on visual assessment, even between highly experienced radiologists [55,56,57]. Within chest CT imaging, reports are often broad and give an overall impression of the disease process. It is either binary (disease present or absent) or a rough categorical assessment of a degree of severity (mild, moderate, or severe). Clinically significant differences have been demonstrated, even in assigning final diagnosis in ILD [58].

QCT, in comparison, provides reproducible data which numerically quantify disease severity [30,59]. It therefore provides an objective measure which can be integrated into diagnostic and prognostic models. This principle of repeatability minimizes the inherent variability from a visual approach to imaging.

#### 4.1.2. Increased Efficiency to Counter Rising Demand

The demand for medical imaging is rising far steeper than the available radiology workforce [30,60,61]. In 2015, it was estimated that an average radiologist must interpret an image every 3–4 s in a normal 8 hour workday to meet workload demands [62]. The demand is particularly steep for complex investigations that require longer to report, such as chest CT and cardiac MRI. These modalities are routinely used in PH and are integral to assessment.

AI has been shown to improve productivity, reducing the time needed to review imaging. Within PAH, routine and repetitive tasks such as measuring pulmonary artery size or right atrial area could be automated, saving time over hand-drawing regions. Temporal subtraction is an AI method to highlight interval change between successive imaging [63,64]. In bone CT scans, this method reduced reading time by 43% and increased sensitivity by 14.6% [65]. PH patients require regular reassessment with imaging to monitor treatment response, and such techniques could help increase the efficiency of repeat comparative reporting.

Triaging of scans is another area which has seen significant research [66]. Scans are first read by the AI model which then categorizes them based on probability of disease. This would enable more efficient use of resources, with radiologists prioritizing those scans that are likely to involve the disease, thereby reducing time to report and enabling clinical decision making.

#### 4.1.3. Novel Methodologies beyond Visual Assessment

A limitation of all current imaging modalities is the inability to visualize and assess the distal pulmonary artery vascular—the region of disease in PAH [29]. In PAH, large pulmonary arteries are known to demonstrate dilation, pruning, and abrupt tapering or tortuosity [67]. Pulmonary vessel morphology is an example of an approach made possible only through quantitative AI analysis, as it would be unfeasible visually. The pulmonary vascular tree is segmented, with features such as lumen size, tortuosity, and tapering quantified. There exist several 3D vessel lumen segmentation techniques in both CT and MRI to enable this [68]. A deep learning CNN approach recently demonstrated 94% accuracy in segmentation of the vascular tree [69]. The findings from clinical applications of such approaches could have an impact on diagnosis and prognostication.

### 4.2. Diagnosis

#### 4.2.1. Reducing Time to Diagnosis and Error

A dual reader approach has been shown to reduce error and misses. However, this is prohibitively time and radiologist resource intensive. AI systems have, therefore, been suggested as an alternative “second reader” and have shown promise in reducing errors and improving sensitivity [64].

Quantifying severity with a continuous index rather than a broad visually estimated category can increase potential to detect more subtle changes [51]. In total, 48% of PH patients do not receive a diagnosis until one year after experiencing symptoms, and 40% see four or more health care providers prior to diagnosis [70]. Chest CT is commonly performed, however, radiologists assessing the lungs do not routinely evaluate for features of PH, and some characteristic abnormalities of CTEPH may be subtle and require specialist radiology expertise limited to tertiary centers. AI models that automatically evaluate studies for signs of PH have the potential to reduce the time to diagnosis.

In emphysema, airway wall thickening visualized on incidental chest CT was shown to be an independent predictor of COPD exacerbations that led to hospitalization or death in a large multicenter randomized controlled trial [71]. A similar simple approach can be applied to PH where QCT could automatically segment, measure, and plot the main pulmonary artery size against a normative curve. This would provide the reporting radiologist with additional context to evaluate and consider PH as a potential diagnosis.

#### 4.2.2. Improving Phenotyping and Classification

In COPD, new subtypes and phenotypes have been discovered through ML approaches [72,73]. These distinct patient subtypes characterized by imaging correlate with physiological parameters. In ILD, QCT metrics have been shown to correlate well with a range of clinical function tests such as lung function tests [52,59,74,75].

PH-Lung has multiple different phenotypes with distinct treatment responses [2,3]. A current clinical dilemma in PH is differentiating IPAH with mild lung disease from PH-Lung; therefore, there is a need to understand more about different phenotypes and why some patients with variable degrees of parenchymal disease do or do not develop PH [5,76]. New phenotypes have also been proposed in IPAH based on patterns of lung involvement on CT [6,11] that differ significantly in treatment response to PAH therapy and prognosis. Quantitative approaches could therefore aid identification of new phenotypes and improve on assessments based on traditional visual based assessment alone.

#### 4.2.3. Discovering Genotype-Phenotype Associations

Four large collaborative genomic and multi-omic programs and biobanks are established for PAH—PVDOMICS (Redefining Pulmonary Hypertension through Pulmonary Vascular Disease Phenomics), US PAH Biobank, and UK national IPAH cohort [9,77,78,79]. Bone morphogenetic receptor type 2 (BMPR2) gene abnormalities are the most common cause of heritable PAH, comprising ~15% of all cases, but 20+ new genes have been identified [9,80]. Patients with BMPR2 mutations are unlikely to demonstrate vasoreactivity, which informs clinical management [81]. Advances in genetic understanding and targeting BMPR2 have developed novel therapies that are tested in clinical trials [82].

The distinct imaging appearances of these phenotypes are not currently well characterized. Cardiac MRI has demonstrated right ventricular (RV) function to be more severely affected in BMPR2 patients, but CT features are currently an area of research [83]. Marrying genetic data with imaging data offers the potential for better phenotype patients.

### 4.3. Prognostication

#### 4.3.1. Predicting Treatment Response and Survival

In idiopathic pulmonary fibrosis (IPF), texture based QCT of lung parenchymal disease patterns was superior to both visual scoring and traditional lung function tests in predicting outcomes [84]. QCT specific lung texture patterns were also found to be an independent predictor for survival when comparing short term interval changes between two scans [85]. For radiologist interpretation, only the overall disease progression was a predictor, and not specific lung features. Models have also demonstrated the ability to identify and select patients who would be steroid responders [86]. In chronic hypersensitivity pneumonitis, extent of fibrosis and reticulation independently predicted time to death or lung transplantation [87]. Another study found severity of traction bronchiectasis and honeycombing to predict mortality [88]. As an example of going beyond conventional metrics such as lung function tests, severity of traction bronchiectasis on HRCT (High-resolution computed tomography) was found to be an independent predictor for mortality in those patients that had marginal annual forced vital capacity (FVC) declines [89].

In COPD, large longitudinal multicenter prospective trials such as COPDGene and SPIROMICS include QCT data to better understand the disease process [90,91]. The results of these studies have found the impact of QCT metrics on a vast range of outcomes [92,93]. In emphysema, lung volume reduction surgery is a treatment in which disease distribution pattern and fissure integrity are important predictors of success [94]. Regional quantification by QCT models here has shown to predict postoperative lung function, thereby aiding clinical decision making [95]. These findings demonstrate the prognostic potential of QCT in PH.

#### 4.3.2. Imaging Biomarkers as Clinical Endpoints

Traditional biochemical biomarkers in PAH include B-type natriuretic peptide (BNP) and N-terminal pro-brain natriuretic peptide, which non-specifically correlate with myocardial function and pulmonary haemodynamics [96]. These are routinely used in practice to inform clinical opinion. 

Imaging biomarkers are imaging features of pathological conditions [97]. Traditional CT imaging biomarkers in PH include mPA size and secondary signs of heart failure such as inferior vena cava dilation, pleural effusions, and septal lines [27]. An mPA size >29 mm has 97% positive predictive value for PH, and a PA to ascending aorta ratio >1 is 92% specific for a raised mean arterial pressure >20 mmHg [29,98,99]. Quantitative CT enables more complex biomarkers that can combine multiple measurements or perform higher level textural analysis to create a model that could be then validated to be diagnostic or prognostic value. While the majority of studies focus currently on the analytical performance of such models, Swift et al. last year validated a CT model against clinical outcomes to demonstrate both diagnostic and prognostic value in suspected PH [28].

Current assessment tools in PH clinics and endpoints used in clinical trials such as the 6 minute walking distance and right heart catheterization are limited, in part, by their insensitivity to change and their invasive nature, respectively [29]. As highlighted by the Pulmonary Vascular Research Institute statement on imaging in PH, there is a need to identify new tools for both clinical use and for use as endpoints in studies [29]. There is particularly an unmet need for biomarkers that can help differentiate between PAH and PH-Lung [17]. Imaging biomarkers identified by QCT metrics may help solve this clinical dilemma.

Repeatable and quantifiable imaging biomarkers can measure treatment response and are currently being used as endpoints for clinical investigations and trials in emphysema and ILD [29,100,101]. QCT metrics have been shown to predict outcomes better than lung function tests in ILD [84,89].

#### 4.3.3. Enabling Precision Medicine and Big Data Analysis

The numerical nature of QCT derived imaging biomarkers naturally integrates well with big data analysis in precision medicine. Precision medicine is a process of enabling targeted tailored therapies to patient groups through deep phenotyping of patients [102]. The goal is bringing together data from genetics, imaging, immunology/histology, and traditional clinical assessment in a holistic manner to refine diagnosis and offer target therapies that improve outcomes. PH as a heterogeneous condition with distinct sub-phenotypes is well suited for precision medicine. A multi-domain and multimodality approach is already established for clinical assessment. The tenth biannual symposium of the International Society for Strategic Studies in Radiology recognized the implementation of quantitative imaging as critical to this goal [103]. It highlighted how imaging findings have strong yet currently untapped potential to guide patient care and influence outcome through imaging-based biomarkers.

## 5. Limitations, Challenges, and Solutions

The promise and the potential of AI should be tempered by a realistic, pragmatic understanding of current limitations. We highlight three limitations to chest QCT and discuss three challenges faced by all ML, radiomic, and imaging biomarker studies.

### 5.1. Limitations in Quantitative CT Research

#### 5.1.1. Variations in Data

A large number of steps exist within the imaging data pipeline, each of which can add variation and noise to the data. These include CT image acquisition parameters, reconstruction, segmentation, feature-extraction and post-processing algorithms. These factors reduce the robustness, performance, and generalisability of radiomics or imaging biomarker approaches, including quantitative CT.

Solutions are therefore an active area of research. The Image Biomarker Standardisation Initiative and groups such as the Quantitative Imaging Biomarker Alliance, and the Association of University Radiologists Research Alliance Quantitative Imaging Task Force are dedicated to such solutions [51,104,105]. These range from standardisation of imaging protocol to more recent algorithms and data processing techniques that limit variance. The SPIROMICS study developed an imaging protocol specifically for Chest QCT [91]. Pyradiomics is a flexible open-source approach to feature extraction, allowing for more widespread reproducible feature analysis [106]. Differences in extracted features between different CT scanners can be tested with physical phantoms to understand the underlying variation [107]. These features can then be standardised amongst protocols to account for this invariance [97,108]. Differences in slice thickness, voxel sizes and convolutional kernels can be normalised using a range of approaches such as voxel-size resampling, batch effect correlation, and grey-level normalisation [109,110,111]. A predictive internal calibration approach was shown to improve performance of emphysema prediction in a COPD study [112]. Moving to an ML based automated approach for segmentation has higher accuracy and reduced variability compared to manual segmentation [113]. For DL approaches, domain adaptation and transfer learning are approaches insensitive to data heterogeneity [97,114,115]. Convolutional neural networks have been shown to dramatically improve the similarity of CT radiomic features obtained with different imaging reconstruction algorithms and kernels [116].

#### 5.1.2. Inspiration, Breathing and Motion Artefacts

Differences in lung volume secondary to inspiratory effort and artefacts from breathing and cardiac motion are inherent to chest CT [117]. They limit traditional visual asessment for radiologists and can lead to misinterpretation, as pulmonary density is influenced by respiratory phase. For a quantitative approach, these must be minimised to avoid errors propagating throughout the pipeline. Failure to do so can lead to errors where the disease is improperly quantified. Solutions include use of modern scanners with rapid acquisition times and larger detectors, clear instructions to patients to explain the importance of breath holding, and data techniques to adjust for variability. Parametric response mapping is a method where volumetric non-rigid registration of both inspiratory and expiratory scans are fused [118]. The overall lung volume can be quantified to alert for differences and used to normalise or weight measurements [119].

#### 5.1.3. Lack of Studies Involving Intravenous Contrast

Most QCT applications to date use non-contrast CT. In PH, most studies involve intravenous contrast and are performed as a CT Pulmonary Angiogram (CTPA). Intravenous contrast increases the opacification of the lung parenchyma due to perfusion of a high density contrast media throughout the pulmonary vessels and lung parenchyma. Whilst the volume and speed of contrast material administered is routinely standardised, there can be variability between scans in the extent of contrast uptake within lung parenchyma. In pulmonary nodule characterization studies, this variability was reduced by acquiring images between 60–150 sec post injection [97,120]. We found only one study assessing lung parenchymal patterns in contrast Chest CT, finding significant difference in mean lung density in patients with pulmonary embolism [121]. This is an area in need of further research. Can the existing algorithms trained on non-contrast CT be applied to CTPA? Can they be adapted using additional information such as density of contrast in main pulmonary arteries and cardiac chambers?

### 5.2. Current Challenges Facing Machine Learning Research

#### 5.2.1. High-Quality Training Data Is Hard to Obtain

Development of any algorithm requires robust training data—both in quantity and quality. The performance of ML models improves logarithmically with increased volume of training data available [122,123,124]. As the algorithm ‘learns’ through feature recognition, the quality of the training cohort fundamentally shapes its performance. An ideal training data set is contextual to the problem it seeks to address, expertly labelled, quality controlled against imaging artefacts and noise, and appropriately powered for its clinical use. It should follow the FAIR principles of scientific data management and stewardship—be Findable, Accessible, Interoperate and Reusable [125]. The lack of high-quality labelled training data is a limitation throughout all domains of ML research. Carefully preparing, validating and labelling training data often form the bulk of the development work [124].

All patient identifiable information needs to be carefully removed from any imaging data set prior to use. Although standards exist for medical imaging data such as DICOM, they are only loosely adhered to, with wide variation in the metadata [124]. Patient information can be difficult to remove, and at times hard coded into the imaging data. Clinical governance standards are stricter for medical data, and mandate secure data management and storage solutions.

To address these challenges, standardised and data validation systems have been proposed. Kohli et al proposed an extensive 16 point baseline metadata list to consider to catalog medical imaging data [126]. A Medical imaging DAta Readiness (MiDAR) scale has been proposed as a four-point framework that assesses the readiness of medical imaging data for development [124]. All AI studies should have a data preparation and quality control framework that ensures training data robustness.

#### 5.2.2. Lack of External Multi Centre Validation and Prospective Studies

ML algorithms should be validated in external multi-centre cohorts to avoid overfitting. Overfitting describes the ML model being exceedingly narrow in its performance, such that it learns from noise and other specific quirks of the training data. Therefore, its performance degrades on external validation cohorts when other variances and variables are present. Overfitting is a major obstacle that hinders generalizability—the application of the clinical model in other similar cohorts or centres. This can be minimised by using a large, diverse training dataset and performing techniques such as augmentation, regularisation and dropout [127].

Of 82 studies describing 147 patient cohorts that compared AI performance vs health care professionals, Liu et al found only 25 performed external validation [41]. Of these, only 14 used the same sample for comparison. Another review found only 9 to be prospective and 6 to be tested in a real world clinical setting [128]. Both reviews found a high risk of potential bias in the validation procedures and poor methodology in study design. These findings highlight the need for further prospective studies designed with external multi-centre validation as a primary target. The need for generalisability inherently has the trade-off for poorer performance across those several centres over stronger performance at a single centre [129]. The retrospective nature of studies leads to large variations in imaging protocols, sample sizes and AI approaches. There is a need for standardisation in reporting practices [130]. In late 2020, SPIRIT-AI (Standard Protocol Items: Recommendations for Interventional Trials-Artificial Intelligence) and CONSORT-AI (Consolidated Standards of Reporting Trials-Artificial Intelligence) standards were published to help improve this in the context of interventions and clinical trials involving AI [131,132].

#### 5.2.3. ‘Black Box’ and Interpretability

DL approaches by design have multiple hidden layers that obscure the decision-making process. This lack of transparency has been labelled a ‘black box’ problem. Approaches are being developed that offer more insight to improve interpretation, such as through visualisation of features [133]. Medical systems and workflows value interoperability as assumptions can be checked and errors appropriately evaluated. There are debates from a legal, regulatory and accountability standpoint regarding the suitability of such systems. Explainable and interpretable AI therefore is a field which is getting more interest. [134]. In the near future, we expect QCT models to serve as an adjunct to visual assessment. Therefore, AI and ML systems which are more clinically comprehensible and better integrated into current clinical imaging workflows will be preferred.

## 6. Conclusions

Leveraging the power of ML has demonstrated significant breakthroughs in a range of lung diseases. These can be applied to improve both radiological assessment and clinical management in pulmonary hypertension. Quantitative imaging in particular can lead to a data-driven decision-making process which combines clinical, physiological, genetic and radiological data for better assessment. This would help answer the topical clinical and radiological problems in the field—helping with better phenotyping and assessment for treatment decisions. Once validated, imaging-based biomarkers can help assess treatment response and therefore can be used as endpoints in clinical trials. The growing use of AI will help reduce errors, increase productivity, and can enable a precision medicine approach to pulmonary hypertension.

It is, however, important to be realistic and pragmatic about the current state of medical imaging using AI, with a clear understanding of its limitations. AI has been shown to excel in the narrow domain sensory image perception and identification tasks. It however does not have the ability to make broader assessments away from its domain, recognise the larger context of its use or even appraise if it is being deployed appropriately. This role falls to humans. Current AI should best be viewed and used as a specific tool in a validated narrow clinical context. Going forward, further prospective, large multi-centre studies are required to better assess technical development in clinical settings. Studies using clinically meaningful endpoints for AI algorithms such as treatment response and survival are preferred over purely technical performance in image classification.

In conclusion, for physicians managing patients with PH and associated lung disease, it is hoped that the application of AI approaches to CT imaging, may “come to the rescue” by providing mechanistic insights and improved phenotyping and by doing so facilitate much needed therapy trials.

## Data Availability

Not applicable.
